# FXYD8, a Novel Regulator of Renal Na^+^/K^+^-ATPase in the Euryhaline Teleost, *Tetraodon nigroviridis*

**DOI:** 10.3389/fphys.2017.00576

**Published:** 2017-08-08

**Authors:** Pei-Jen Wang, Wen-Kai Yang, Chia-Hao Lin, Hau-Hsuan Hwang, Tsung-Han Lee

**Affiliations:** ^1^Department of Life Sciences, National Chung Hsing University Taichung, Taiwan; ^2^Department of Public Affairs and Civic Education, National Changhua University of Education Changhua, Taiwan; ^3^Bachelor Degree Program in Animal Healthcare, Hungkuang University Taichung, Taiwan; ^4^National Institute for Basic Biology, National Institutes of Natural Sciences Okazaki, Japan; ^5^Agricultural Biotechnology Center, National Chung Hsing University Taichung, Taiwan

**Keywords:** FXYD8, Na^+^/K^+^-ATPase, kidney, pufferfish, *Tetraodon nigroviridis*, salinity

## Abstract

FXYD proteins are important regulators of Na^+^/K^+^-ATPase (NKA) activity in mammals. As an inhabitant of estuaries, the pufferfish (*Tetraodon nigroviridis*) responds to ambient salinity changes with efficient osmoregulation, including alterations in branchial, and renal NKA activities. Previous studies on teleostean FXYDs have mainly focused on the expression and potential functions of FXYD proteins in gills. The goal of the present study was to elucidate the potential role of FXYD8, a member of the fish FXYD protein family, in the modulation of NKA activity in the kidneys of this euryhaline pufferfish by using molecular, biochemical, and physiological approaches. The results demonstrate that *T. nigroviridis* FXYD8 (TnFXYD8) interacts with NKA in renal tubules. Meanwhile, the protein expression of renal TnFXYD8 was found to be significantly upregulated in hyperosmotic seawater-acclimated pufferfish. Moreover, overexpression of TnFXYD8 in *Xenopus* oocytes decreased NKA activity. Our results suggest the FXYD8 is able to modulate NKA activity through inhibitory effects upon salinity challenge. The present study further extends our understanding of the functions of FXYD proteins, the regulators of NKA, in vertebrates.

## Introduction

Na^+^/K^+^-ATPase (NKA) is a ubiquitous membrane-bound protein complex that actively maintains the Na^+^ and K^+^ gradient between the intra- and extracellular milieu of animal cells, and plays a crucial role in osmoregulation and ion exchange (Scheiner-Bobis, 2002; Marshall and Grosell, 2006; Hwang and Lee, 2007). It is a P-type ATPase consisting of an (αβ)_2_ protein complex (Scheiner-Bobis, 2002; Toyoshima et al., 2011). The molecular weights of the catalytic α-subunit and the smaller glycosylated β-subunit are about 100 and 55 kDa, respectively (Scheiner-Bobis, 2002). NKA facilitates many transportation systems in order to sustain homeostasis (Scheiner-Bobis, 2002; Hwang and Lee, 2007; Suhail, 2010). Hence, modulation of the activity or kinetics of NKA is essential for cellular physiological functions to be performed (Marshall and Grosell, 2006; Hwang and Lee, 2007; Whittamore, 2012). Several mechanisms have been reported to be involved in the regulation of NKA activity, including the FXYD proteins (Scheiner-Bobis, 2002; Crambert and Geering, 2003; Geering, 2008).

The FXYD proteins (also called FXYD domain-containing ion transport regulators) are named based on their invariant extracellular FXYD motif, and belong to a family of proteins containing a single conserved transmembrane domain (Sweadner and Rael, 2000; Crambert and Geering, 2003; Garty and Karlish, 2006). These proteins possess similar amino acid sequences, including a conserved FXYD motif, two identified glycine residues, and a serine residue (Crambert and Geering, 2003; Garty and Karlish, 2006). Seven members of the FXYD protein family (i.e., FXYD1–7) exist in mammals, and they all exhibit distinct tissue-specific distributions (Crambert and Geering, 2003; Geering, 2008). FXYD10, of the elasmobranch, was the first reported FXYD family member in fish (Mahmmoud et al., 2000). Recently, several FXYD proteins (i.e., FXYD2, 5–9, 11, and 12) were identified in teleosts (Tipsmark, 2008; Wang et al., 2008; Saito et al., 2010; Tang et al., 2012; Yang et al., 2013; Hu et al., 2014). Similar to mammalian FXYD proteins, teleostean FXYD proteins were also found to be distributed in a tissue-specific manner (Tipsmark, 2008; Saito et al., 2010; Tang et al., 2012; Yang et al., 2013; Hu et al., 2014).

Euryhaline teleosts can adapt to a broad range of ambient salinities and maintain their internal osmolality within a fixed range (Hwang and Lee, 2007; Whittamore, 2012). Salinity acclimation of euryhaline teleosts is a complex process involving a series of physiological responses by osmoregulatory organs, including the gill, kidney, and gut, to milieus' with differing osmoregulatory requirements (Marshall and Grosell, 2006; Hwang and Lee, 2007; Whittamore, 2012). In freshwater (FW) teleosts, the hypotonic environment results in the diffusional loss of ions and the osmotic influx of water. Ionic homeostasis is thus maintained through the absorption of ions in the gills and guts, the excretion of excess water, and the reabsorption of filtered solutes in the kidneys of teleosts. In contrast, seawater (SW) teleosts are hypotonic to their environments and passively lose water and gain excess ions. For compensation, they drink seawater and actively excrete most monovalent ions and small amounts of divalent ions, through the gills and kidneys, respectively (Marshall and Grosell, 2006; Hwang and Lee, 2007; Whittamore, 2012). In these processes, renal NKA plays an important role in the facilitation of ionic and osmotic gradients necessary for ion uptake, excretion, and general cellular homeostasis (Marshall and Grosell, 2006; Whittamore, 2012). Salinity-dependent changes in renal NKA activity have been reported in different euryhaline species (Lin et al., 2004; Duffy et al., 2011; Tang et al., 2012; Yang et al., 2016a). On the other hand, only a few studies have investigated the mRNA and protein expression of FXYD proteins in the modulation of renal NKA activity in euryhaline teleosts (Tang et al., 2012; Hu et al., 2014; Yang et al., 2016b). Salinity-dependent mRNA and/or protein expression of FXYD proteins in the kidneys of these euryhaline teleosts is suggestive of their physiological significance in ionoregulation/osmoregulation for osmoregulatory acclimation (Yang et al., 2016b).

The pufferfish (*Tetraodon nigroviridis*) is an advanced tetraodontid teleost, and its native habitats range from rivers (FW) to estuaries (brackish water, BW) (Rainboth, 1996). This pufferfish, being a peripheral FW inhabitant, has been demonstrated to be an efficient osmoregulator, because it can tolerate being transferred directly from FW to SW and *vice versa* (Lin et al., 2004; Lin and Lee, 2016). The pufferfish has a genome of 350 Mb, smaller than most vertebrates (Crollius and Weissenbach, 2005), and genetic information is available from the puffer genome database (Genoscope; http://www.genoscope.cns.fr/externe/tetranew/). Although the entire pufferfish genome has not been sequenced, it has contributed to molecular investigations in teleosts. Its euryhalinity, wide availability, and inexpensive maintenance make the pufferfish a good experimental model for use in studies on osmoregulation/ionoregulation (Wang et al., 2008). In pufferfish gills, the expression of NKA and FXYD9 was investigated following salinity challenges (Lin et al., 2004; Wang et al., 2008; Lin and Lee, 2016). Moreover, the salinity-dependent response of the two proteins, as well as their interaction, showed that the pufferfish FXYD protein might play important roles in osmoregulation via the modulation of NKA expression as mammalian FXYD (Wang et al., 2008). On the other hand, differences in protein abundance, as well as in the activity of renal NKA, were found between FW- and SW-acclimated pufferfish (Lin et al., 2004). In response to changing salinities in the estuary, pufferfish must have a strategy for efficient ionic regulation and acclimation. The expression and function of NKA regulators, such as FXYD proteins, in the kidneys of the euryhaline pufferfish are therefore worth investigation.

The expression and functions of most FXYD proteins in mammals and elasmobranches have been widely studied (Garty and Karlish, 2006; Geering, 2008). Moreover, to date, most studies on teleostean FXYD proteins have focused on certain FXYD members in gills of limited species (Saito et al., 2010; Tipsmark et al., 2010, 2011; Yang et al., 2013). In kidney (another osmoregulatory organ), very little is known about the expression and functions of teleostean FXYD proteins. To elucidate the regulatory mechanisms of renal NKA activity in pufferfish with efficient responses to ambient salinity challenge, we aimed to investigate patterns of *T. nigroviridis* FXYD8 (TnFXYD8) mRNA/protein expression. FXYD8 is a novel member of the FXYD protein family in euryhaline teleosts, and this study investigated the localization and interaction between TnFXYD8 and NKA in the kidneys of pufferfish acclimated to FW and SW. The role of pufferfish FXYD8 in the modulation of NKA activity was also determined. This is the first study to explore the physiological regulation of teleostean FXYD8 protein and demonstrate its effect on NKA activity using an *in vitro* overexpression system. The findings of this study will further extend our understanding about the potential roles of FXYD proteins in regulating NKA activity in the fish kidney.

## Methods

### Experimental animals

Pufferfish (*T. nigroviridis*; 4–9-g body weight; 4–5-cm total length) was obtained from a local aquarium. Fish were reared in either SW or FW at 27 ± 1°C with a daily 12-h photoperiod for at least 4 weeks before sampling. Environmental conditions and animal care procedures have been described previously (Lin et al., 2004; Wang et al., 2008). Fish were not fed and were anesthetized with MS-222 (100–200 mg/L) before sampling. The protocols employed for the experimental fish were reviewed and approved by the Institutional Animal Care and Use Committee of the National Chung Hsing University (IACUC approval no. 95–82), and were carried out in accordance with the approved guidelines.

### Total RNA extraction and reverse transcription

The methods used herein were modified from those described by Yang et al. (2013). To determine the abundance of *Tnfxyd8* mRNA, total RNA was extracted from the whole kidney and purified using the RNA-Bee isolation kit (Tel-Test, Friendwood, TX, USA) and RNAspin Mini kit (GE Health Care, Piscataway, NJ, USA), respectively, following the manufacturer's instructions. RNA integrity was verified by 0.8% agarose gel electrophoresis. Extracted RNA samples were stored at −80°C after isolation. For reverse transcription, first-strand cDNA was synthesized using SuperScript™ Reverse Transcriptase (Invitrogen, Carlsbad, CA, USA) following the manufacturer's instructions. The cDNA products were stored at −20°C until analysis via polymerase chain reaction (PCR).

### TnFXYD8 sequences

The full-length TnFXYD8 DNA sequence (HM585097) was verified by PCR and DNA sequencing, and then uploaded to NCBI GenBank (http://www.ncbi.nlm.nih.gov/). To clone the full-length TnFXYD8 cDNA, FINNZYMES Phusion High-Fidelity PCR kit (NEB, Ipswich, MA, USA) was used following the manufacturer's manual. For the RT-PCR amplification (35 cycles), 1 μL cDNA was used as a template in a 25-μL final reaction volume containing 0.25 μM dNTPs, 1.25 U Hot start EX-Taq polymerase (Takara, Shiga, Japan), and 0.5 μM primer. The PCR cycle protocol was 95°C for 1 min, 30 cycles of 95°C for 1 min, 53°C for 90 s, and 72°C for 2 min, with a final incubation at 72°C for 15 min. All primers are listed in Table S1. The PCR product was stored at 4°C before being run on 1% agarose gel. PCR products were subcloned into the pOSI-T vector (Genemark, Taipei, Taiwan), and amplicons were sequenced for confirmation.

To characterize the TnFXYD8 sequence, nucleotide consensus sequences were translated to protein using the translate resource at the ExPASy proteomics server (http://www.expasy.org/sprot/). Afterwards, transmembrane segments and signal peptides were predicted on the TMHMM 2.0 (http://www.cbs.dtu.dk/services/TMHMM-2.0/) and SignalP 3.0 servers (http://www.cbs.dtu.dk/services/SignalP/), respectively. Potential phosphorylation and *O*-glycosylation sites were predicted by the NetPhos 2.0 (http://www.cbs.dtu.dk/services/NetPhos/) and NetOglyc 3.1 servers (http://www.cbs.dtu.dk/services/NetOGlyc/), respectively.

### Phylogenetic analysis

Published sequences of teleost FXYD proteins (Tipsmark, 2008; Wang et al., 2008; Saito et al., 2010; Tang et al., 2012; Yang et al., 2013; Hu et al., 2014) were obtained from NCBI. All accession numbers are listed in the Table S2. Multiple sequence alignments were performed by Bioedit version 7.2.5 with ClustalW (Hall, 1999). The phylogenetic tree was constructed using MEGA7 with the maximum likelihood method (Kumar et al., 2016), and 1,000 bootstraps were used to test the consistency of the groupings.

### Real-time PCR

Real-time PCR analysis and calculation of target gene expression following the methods described previously (Wang et al., 2008; Kang et al., 2013) with modifications. Each PCR contained 8 μL cDNA (100 × dilution), 2 μL of either *Tnfxyd8* or β*-actin* (internal control) primer mixture (100 nM), and 10 μL SYBR Green PCR Master Mix (Applied Biosystems, Foster City, CA, USA), using the ABI PRISM 7300 Real-Time PCR System (Applied Biosystems). Primer sequences are shown in Table S1. Melting curve analysis and electrophoresis were performed to confirm the specificity of the amplification. For each unknown sample, relative *Tnfxyd8* expression was obtained using the formula 2^∧^ −[(Ct_*Tnfxyd*8__,*n*_−Ct_β−*actin*__,*n*_)−(Ct_*Tnfxyd*8__,*c*_−Ct_β−*actin*__,*c*_)], where Ct corresponded to the threshold cycle number.

### Antiserum/antibody

The primary antisera/antibodies used in this study included: (1) TnFXYD8: rabbit polyclonal antiserum against the specific epitope (ASADDKDYDSDFYYDYKS) corresponding to the N-terminal region of the cloned TnFXYD8 (Yao-Hong Biotechnology, Taipei, Taiwan); (2) NKA: mouse monoclonal antibody (α5; Developmental Studies Hybridoma Bank, Iowa City, IA, USA) raised against the α-subunit of avian NKA; and (3) actin: mouse monoclonal antibody (MAB1501; Millipore, Billerica, MA, USA) raised against chicken gizzard actin, used as a loading control for immunoblotting (Wang et al., 2008). Secondary antibodies used for immunoblotting were HRP-conjugated goat anti-mouse IgG or goat anti-rabbit IgG (#0031430 or #0031460, respectively; Pierce, Rockford, IL, USA).

### Immunoblotting

Tissue homogenates were prepared as described by Yang et al. (2016a). After homogenization and centrifugation, renal supernatants were collected and processed immediately to determine protein concentration or stored at −80°C for immunoblotting. Protein concentrations were identified using the Protein Assay Kit (Bio-Rad, Hercules, CA, USA), with BSA (Sigma) as a standard.

Immunoblotting was performed as previously described (Wang et al., 2008; Yang et al., 2011) with some modifications. Briefly, renal lysates were heated at 100°C for 5 min or at 37°C for 30 min (for TnFXYD8 or NKA, respectively), followed by 15% (for TnFXYD8) or 7.5% (for NKA) SDS-PAGE. Following electrophoresis, proteins were transferred to PVDF membranes (Millipore), and the blots were pre-incubated at room temperature for 1 h in PBST (PBS with 0.05% Tween 20) containing 5% (w/v) non-fat dried milk to minimize non-specific binding. Then, the blots were incubated at room temperature for 1 h with either TnFXYD8 (200,000 × dilution), NKA (2,500 × dilution), or actin (5,000 × dilution) in 5% (w/v) non-fat dried milk and 0.05% sodium azide (Sigma) in PBST, followed by incubation at room temperature for 1 h with a secondary antibody diluted in PBST. Blots were developed with an ECL kit (#34082; Pierce). Immunoblots were photographed and imported as JPEG files into the image analysis software package (MCID 7.0; Imaging Research, Ontario, Canada). Results were converted to numerical values to permit comparison of immunoreactive band intensities. The renal homogenates from FW-acclimated pufferfish were used as the internal control among different immunoblots (data not shown). The intensity of the immunoreactive band of the internal control in the immunoblot was set to one and used as a standard for normalizing relative intensities of other bands in the immunoblots. Relative abundance of target proteins was calculated using the following formula (TnFXYD8_*n*_/actin_*n*_)/(TnFXYD8_*internal control*_/actin_*internal control*_) (Chang et al., 2016a,b).

### Co-immunoprecipitation (Co-IP)

The methods used in this study to prepare renal crude membrane fractions were modified from our previous studies (Chang et al., 2016a; Hu et al., 2017). After homogenization and centrifugation, the supernatants were collected and used to determine protein concentration or to do IP. Protein concentrations were determined as the above description. The co-IP procedure was performed as described by Yang et al. (2016b). Briefly, IP with the anti-TnFXYD8, PBS (negative control), or the anti-NKA was carried out using the ImmunoCruz™ IP/WB Optima System (sc-45042 or sc-45043; Santa Cruz Biotechnology, Santa Cruz, CA, USA) according to the manufacturer's manual. After elution, the samples were stored at 4°C until use. To confirm the interaction between TnFXYD8 and NKA, the obtained IP solutions were analyzed by immunoblotting.

### Overexpression of TnFXYD8 protein in *Xenopus* oocytes

The protocols used herein were modified from previous study (Tseng et al., 2011). pCS2+ vectors (constructed by David L. Turner, Hutchinson Cancer Research Center, and R. A. W. Rupp) were used to overexpress the *Tnfxyd8* gene in *Xenopus* oocytes. To generate the pCS2+*Tnfxyd8* construct, the corresponding TnFXYD8 coding sequence was PCR-amplified using the plasmid pOSI-*Tnfxyd8* (pOSI-T vector carrying the *Tnfxyd8* gene) as the template with the primers: TnFXYD8, 5′-CGGAATTCATGGACCTCGTGGTGTTTGT-3′ (forward) and 5′-GCTCTAGACTATTCTGCTTTGACAGAATAAGG-3′ (reverse). These sequences were then cloned into a pCS2+ vector at the *Eco*RI and *Xba*I sites. *Eco*RI, *Not*I, and *Xba*I restriction sites are underlined. The sequences of all constructs were confirmed by DNA sequencing.

All constructs cloned in the pCS2+ vector were linearized by SacII (Promega, Madison, WI, USA), and the capped RNA was transcribed using a SP6 message RNA polymerase kit (Ambion, Austin, TX, USA). Stage V–VI oocytes were obtained from *Xenopus laevis* as previously described (Dumont, 1972). We injected, *in vitro*, synthesized cRNAs encoding *Tnfxyd8* into oocytes at 8 ng/oocyte in 50 nL Barth's medium (88 mM NaCl, 1 mM KCl, 2.4 mM NaHCO_3_, 0.33 mM Ca[NO_3_]_2_, 0.41 mM CaCl_2_, 0.82 mM MgSO_4_, 15 mM Hepes, pH 7.6; with 1 M NaOH to which the gentamicin [0.1 mg/mL] was added). Oocytes were incubated in Barth's medium at 16°C for 2 days.

### Preparation of *Xenopus* oocyte microsomes

To prepare microsomes, 20 oocytes were homogenized as described previously (Tseng et al., 2011; Yang et al., 2016a). Homogenates were first centrifuged twice at 1,000 × g for 10 min at 4°C to remove yolk granules and then at 10,000 × g for 20 min to yield a microsomal pellet in which 2–3 μg protein/oocyte was recovered. Following the above methods, a previous study revealed that >90% of the total α and β subunits from cRNA-injected oocytes were recovered in microsomal pellets (Geering et al., 1996). The microsomal pellets were dissolved in homogenization mixture (10 μL/oocyte) and then were used to detect endogenous NKA of *Xenopus* oocytes using a NKA antibody (Figure S1) or to perform NKA activity assay.

### NKA activity assay

Endogenous NKA activity in *Xenopus* oocytes was measured by NADH-linked methods (Yang et al., 2011, 2016b). The microsomes of *Xenopus* oocytes were prepared as previously described. NKA activity was calculated as the difference in the slope of ATP hydrolysis (NADH reduction) in the presence and absence of ouabain (1 mM) (Geering et al., 1996), and the activity was expressed as μmol ADP/mg protein/h.

### Statistical analyses

Values are expressed as mean ± standard error. Results were analyzed via Mann-Whitney *U* test (for experiments on the effects of salinity) or Tukey's multiple-comparison test following one-way ANOVA (for overexpression experiments), and *P* < 0.05 was set as the level of significance.

## Results

### Sequence characteristics and phylogenetic analysis of TnFXYD8

**Figure 1:**
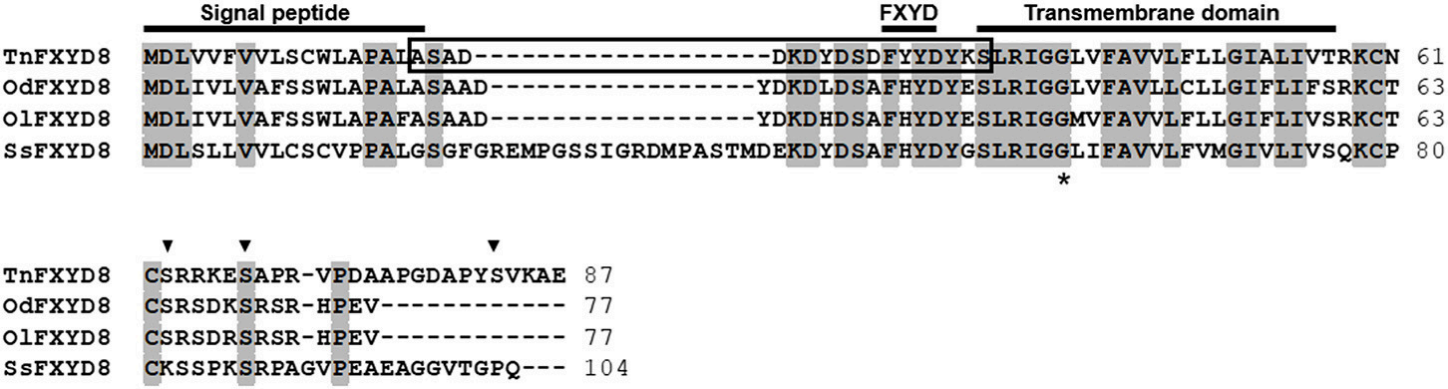
Alignment of amino acid sequences of teleostean FXYD8 proteins. The frame, epitope for specific antibody preparation; black triangle, potential phosphorylation site for TnFXYD8; asterisk, conserved glycine residue (G40); gray background, identical amino acids. All accession numbers are listed in Table S2. Od, brackish medaka (*Oryzias dancena*); Ol, Japanese medaka (*Oryzias latipes*); Ss, Atlantic salmon (*Salmo salar*); Tn, pufferfish (*Tetraodon nigroviridis*).

**Figure 2:**
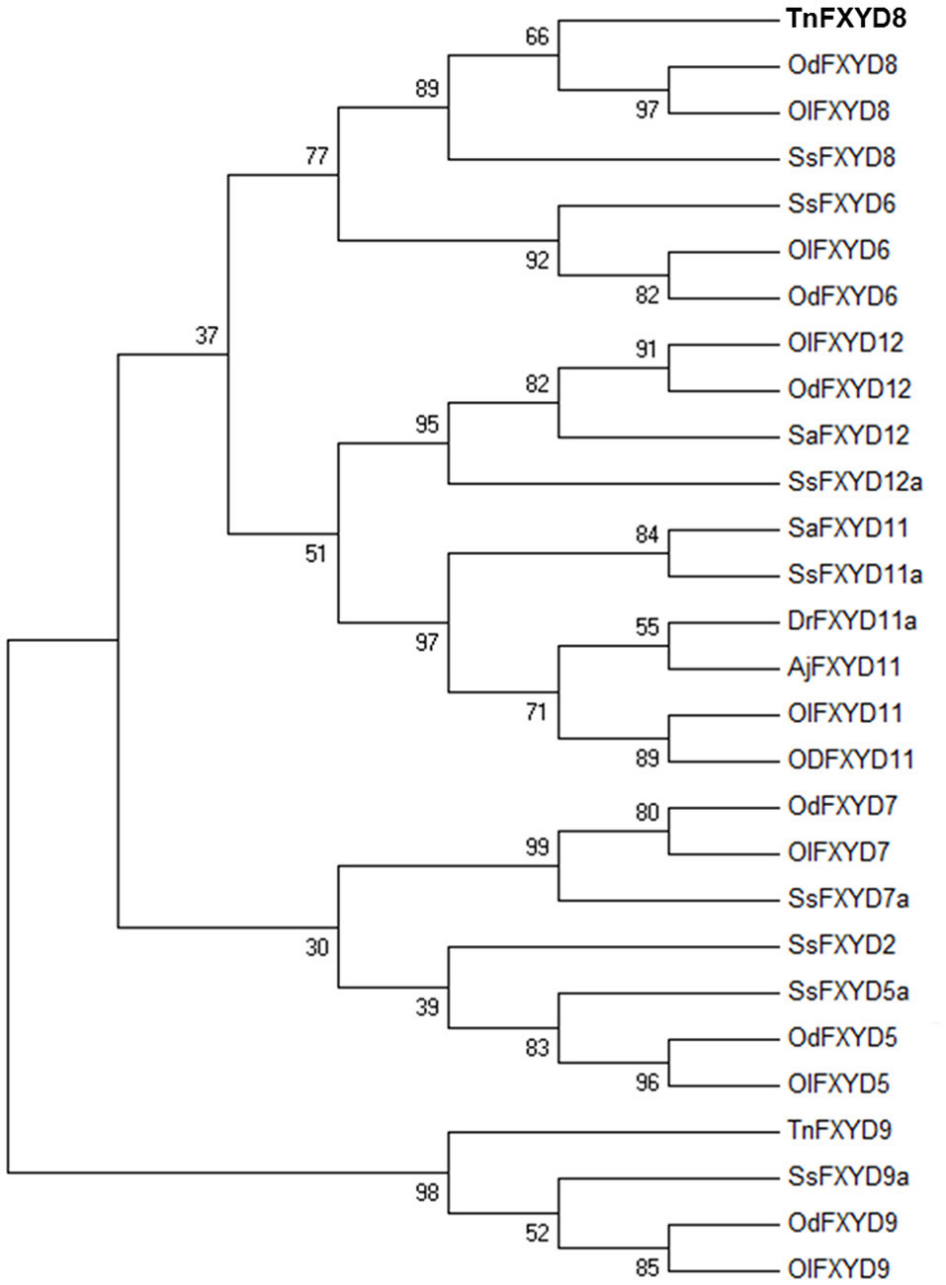
Phylogenetic tree of TnFXYD8 with known FXYD members from various teleosts. Numbers represent bootstrap values for the percentage of 1,000 replicates. All accession numbers are listed in Table S2. Aj, Japanese eel (*Anguilla japonica*); Dr, zebrafish (*Danio rerio*); Od, brackish medaka (*Oryzias dancena*); Ol, Japanese medaka (*Oryzias latipes*); Sa, spotted scat (*Scatophagus argus*); Ss, Atlantic salmon (*Salmo salar*); Tn, pufferfish (*Tetraodon nigroviridis*).

The full-length TnFXYD8 protein consists of 87 amino-acid residues (Figure 1). Based on hydropathy analysis, TnFXYD8 was found to contain one transmembrane domain (residues 35–57), and the first 18 amino acids were predicted as the signal peptide. Three putative phosphorylation sites (black triangles) were identified in the intracellular domain of TnFXYD8, and no glycosylation site was found (Figure 1). Based on multiple alignment with other teleostean FXYD8 proteins, TnFXYD8 was found to be a small protein containing a FXYD motif and one glycine residue (G40; asterisk) at the conserved transmembrane domain. The frame revealed the epitope required for TnFXYD8 antiserum preparation (Figure 1). On the other hand, the phylogenetic tree of the teleostean FXYD proteins showed that TnFXYD8 is closely related to FXYD proteins of two medakas and Atlantic salmon (*Salmo salar*) (Figure 2). Additionally, FXYD8 proteins were grouped with FXYD6 proteins.

### Tissue distribution of *Tnfxyd8* mRNA

**Figure 3:**
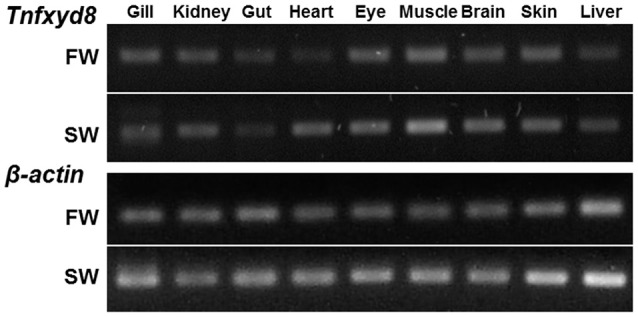
Expression of TnFXYD8 in various tissues of fresh water (FW)- and seawater (SW)-acclimated pufferfish by RT-PCR analysis. The *Tnfxyd8* gene was found in the gill, kidney, gut, heart, eye, muscle, brain, skin, and liver of the pufferfish acclimated to either FW or SW. β*-actin* was used as an internal control.

Reverse transcription-polymerase chain reaction (RT-PCR) analysis, followed by electrophoresis, characterized the tissue-specific expression pattern of *Tnfxyd8* mRNA in FW- and SW-acclimated pufferfish (Figure 3). PCR amplification yielded a band of the predicted size (146 bp) in all studied organs including the gill, kidney, gut, heart, eye, muscle, brain, skin, and liver of pufferfish acclimatized to either FW or SW. PCR products were confirmed to be *Tnfxyd8* cDNA fragments by subcloning and sequencing (data not shown). β*-actin* was also cloned as an internal control to confirm cDNA quality.

### Association of TnFXYD8 with NKA in the pufferfish kidney

**Figure 4:**
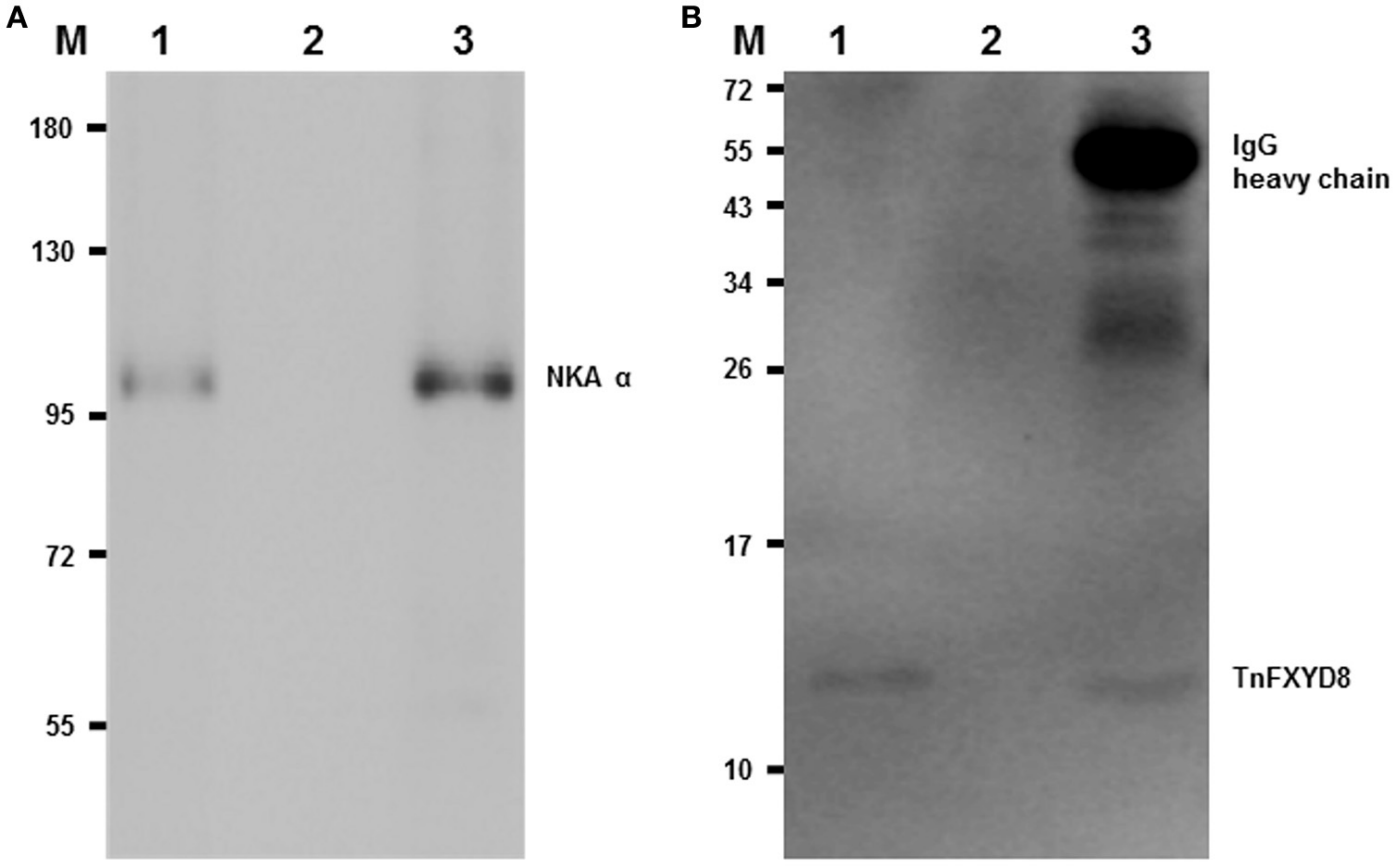
Co-immunoprecipitation (Co-IP) of TnFXYD8 and NKA α-subunit (NKA α) in the pufferfish kidneys. TnFXYD8 or NKA were immunoprecipitated from renal crude membrane fractions of freshwater pufferfish using primary antibodies, then the immune complexes were analyzed by immunoblotting for NKA α **(A)** or TnFXYD8 **(B)**, respectively. In immunoprecipitated NKA α or TnFXYD8, the immunoblotting analyses for NKAα and TnFXYD8 revealed immunoreactive bands at 100 kDa **(A)** and 13 kDa **(B)**, respectively. In (**B)**, the 55-kDa band in lane 3 is the IgG heavy chain of TnFXYD8 antibody. M, marker (kDa); lane 1, immunoblot detection of the opposing antibody (experimental group); lane 2, negative control for no antibody incubation in IP; lane 3, positive control using the same antibody with IP.

To examine the interaction between TnFXYD8 and NKA proteins, immunoprecipitated TnFXYD8 was probed for in complexes of immunoprecipitates for NKA α-subunit and *vice versa* using immunoblotting assays. Indeed, NKA was detected by immunoblotting in immunoprecipitates of TnFXYD8 and the NKA α-subunit (lane 1 and 3 of Figure 4A, respectively), and TnFXYD8 was detected by immunoblotting in immunoprecipitates of the NKA α-subunit and TnFXYD8 (lane 1 and 3 of Figure 4B, respectively). When TnFXYD8 and the NKA α-subunit were immunoprecipitated, bands were apparent at 100 kDa (lane 1 of Figure 4A) and 13 kDa (lane 1 of Figure 4B) corresponding to the molecular weights of the pufferfish NKA α-subunit and FXYD protein, respectively. Lane 2 of Figure 4 contains the negative control, in which no antibody was used to treat immunoprecipitates. Lane 3 of Figure 4 contains the positive control (using the same antibody with immunoprecipitate), which demonstrates the efficiency of the immunoprecipitate. On the other hand, frozen sections of SW pufferfish kidneys dual immunostained with an antibody specific to NKA α-subunit and antiserum to TnFXYD8 were visualized under a confocal microscope (Supplementary Methods). Confocal micrographs (Figure S2) revealed that TnFXYD8 (red cells) was co-localized with NKA-immunoreactive (NKA-IR) cells (green cells), with merged images showing yellow in the renal tubules. Taken together, these data demonstrate that TnFXYD8 interacts with NKA in pufferfish renal tubules.

### Effect of salinity on the expression of renal TnFXYD8 in the pufferfish

**Figure 5:**
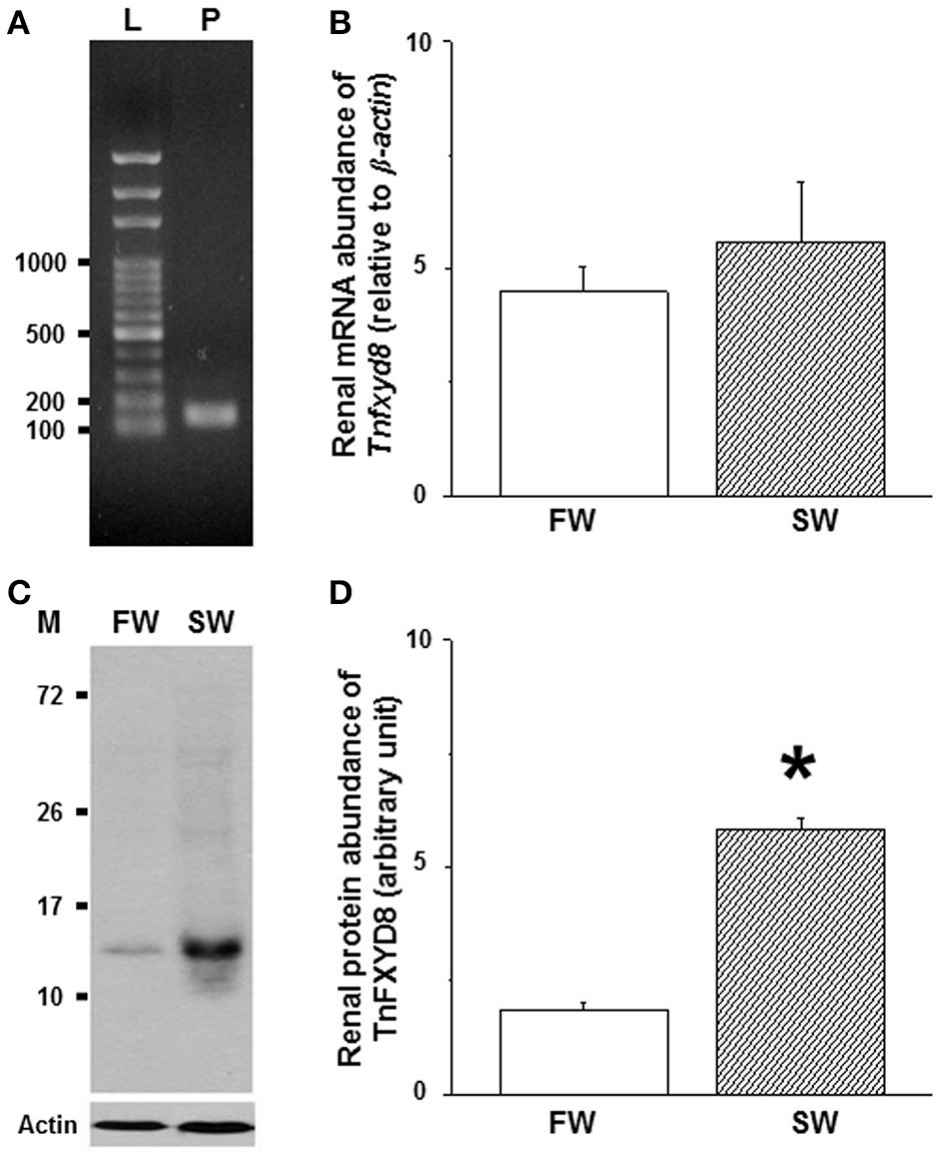
Effects of salinity on mRNA levels **(A,B)** and protein abundance **(C,D)** of renal TnFXYD8 in the pufferfish. **(A)** RT-PCR analysis confirmed primer specificity. **(B)** Comparisons of renal mRNA abundance between freshwater (FW)- and seawater (SW)-pufferfish quantified by real-time PCR. **(C)** Renal protein lysates revealed immunoreactive bands at 13 kDa. **(D)** The relative abundance of TnFXYD8 protein expressed in kidneys of pufferfish acclimated to FW was significantly lower than that in kidneys of the SW group. β*-actin* and actin were used as internal controls for mRNA and protein analyses, respectively. Values are presented as means ± standard error (*N* = 6 or 5 for mRNA or protein, respectively). The asterisk indicates a significant difference (*P* < 0.05) via Mann-Whitney *U* test. L, ladder (bp); M, marker (kDa); P, PCR production.

To quantify the abundance of *Tnfxyd8* mRNA, the specificity of real-time PCR primer was checked by RT-PCR and a 146-bp single band was detected (Figure 5A). No significant difference in *Tnfxyd8* mRNA level was observed between the FW- and SW-acclimated pufferfish (Figure 5B).

To confirm the specificity of TnFXYD8 antiserum, TnFXYD8 protein was overexpressed in bacteria (Supplementary Methods). TnFXYD8 protein was successfully overexpressed and detected by immunoblotting (Figure S3). An additional immunoreactive band was detected at 17 kDa in lysates of bacteria overexpressing TnFXYD8 compared with the non-induced bacteria lysate (Figure S4A). Because the overexpressed protein contained an additional peptide sequence of about 3.8 kDa, which was higher than the predicted molecular weight of TnFXYD8, the actual size of TnFXYD8 was determined to be 13 kDa. The negative control experiment, in which rabbit pre-immune serum replaced TnFXYD8 antiserum, revealed no immunoreactivity in FW- and SW-acclimated pufferfish kidneys by immunoblotting (Figure S4B). Those results showed that the TnFXYD8 antiserum could be successfully applied to detect TnFXYD8 proteins in pufferfish. Further comparisons between FW- and SW-acclimated pufferfish in terms of the relative abundance of renal TnFXYD8 protein were made using actin as a loading control. Immunoblots of renal total lysates from either FW- or SW-acclimated pufferfish revealed a single immunoreactive band for TnFXYD8 (Figure 5C). There was a significant 3.1-fold increase in the intensity of immunoreactive bands for TnFXYD8 in SW-acclimated fish compared with FW-acclimated fish (Figure 5D).

### Functional analysis of TnFXYD8

**Figure 6:**
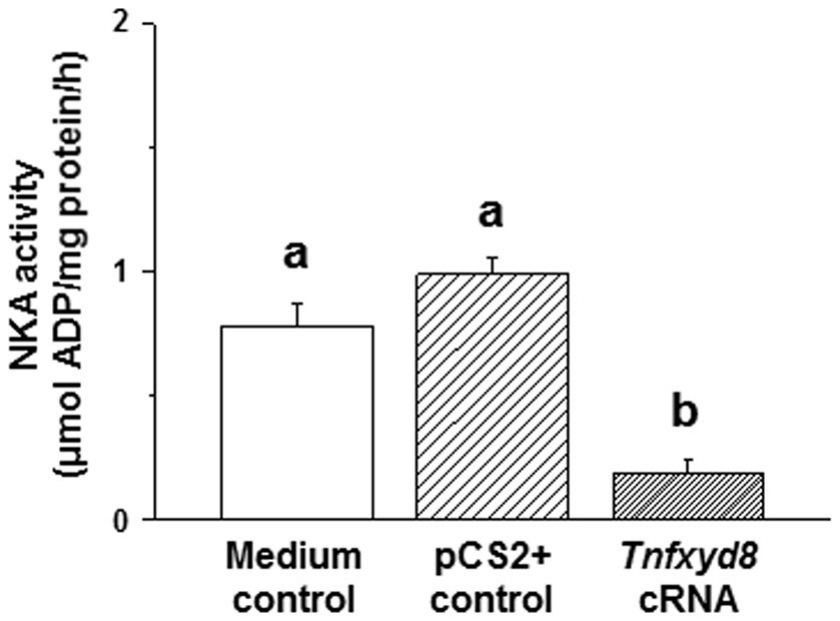
*Xenopus* oocyte Na^+^/K^+^-ATPase (NKA) activity in a TnFXYD8 overexpression experiment. *Xenopus* oocytes from the medium control and pCS2+ control groups were injected with equal volumes of Barth medium and the equal abundance (8 ng) of empty pCS2+ vector, respectively. NKA activity of the TnFXYD8-injected group (*Tnfxyd8* cRNA) was significantly lower than that of the other two control groups. Values are means ± standard error (*N* = 6). Dissimilar letters indicate significant differences among various groups (*P* < 0.05).

To determine whether pufferfish TnFXYD8 homologs are able to modulate NKA activity, heterologous expression studies were conducted in *Xenopus* oocytes. The experimental oocytes were injected *in vitro* with 8 ng synthesized *Tnfxyd8* cRNA, 8 ng (equal abundance) empty pCS2+ vector (pCS2+ control), or 50 nL (equal volume) Barth medium (medium control). NKA activity in the TnFXYD8-injected group was significantly lower than that in both control groups (4.3- and 5.5-fold lower than the medium control and pCS2+ control, respectively; Figure 6). No difference was found between the other two control groups. These *in vitro* results demonstrated that TnFXYD8 was able to inhibit NKA activity.

## Discussion

The FXYD protein family consists of at least 12 members in mammals, elasmobranches, and teleosts (Garty and Karlish, 2006; Hwang et al., 2011). In addition to modulating NKA activity, FXYD proteins were also found to play different roles in mammals, e.g., regulating heart contractility and brain ventricle size as well as expression of sodium/calcium exchanger (FXYD1; Cheung et al., 2007; Geering, 2008; Chakraborti et al., 2016) and affecting formation of tight and adherent junctions to change epithelial morphology (FXYD5; Lubarski Gotliv, 2016). The effects of these FXYD proteins on NKA activity are diverse (Garty and Karlish, 2006; Geering, 2008). In mammals, FXYD1–3 and FXYD7 were reported to negatively regulate NKA activity (Béguin et al., 2001; Crambert and Geering, 2003; Crambert et al., 2005), while FXYD4 and FXYD5 were found to stimulate the activity of mammalian NKA (Garty et al., 2002; Lubarski et al., 2005). Meanwhile, human FXYD8 genes was discovered from on the X chromosome (Davidow et al., 2007) and nothing known about its expression and function (Studer et al., 2011). FXYD8 was postulated to be a potential pseudogene (Studer et al., 2011; Pirkmajer et al., 2017) and needed to be elucidated in further studies. Compared to the mammalian FXYD proteins, the roles of most fish FXYD proteins are unknown to date. In teleosts, salinity-dependent expression has only been investigated in depth for FXYD9, FXYD11, and FXYD12, and their potential roles in osmoregulatory organs have been illustrated (Wang et al., 2008; Tipsmark et al., 2010, 2011; Tang et al., 2012; Yang et al., 2013, 2016b; Hu et al., 2014; Chang et al., 2016b). Hence, the present study investigated the expression and function of TnFXYD8 in pufferfish kidneys to illustrate the mechanisms underlying the regulation of NKA activity in euryhaline teleosts.

In this study, the cloned TnFXYD8 protein was found to contain a highly conserved FXYD motif and a conserved transmembrane domain in common with vertebrate FXYD proteins (Garty and Karlish, 2006; Geering, 2008). These conserved residues were considered to be involved in the function of the protein, including the structural interaction with NKA (Béguin et al., 2001; Geering, 2005; Garty and Karlish, 2006). Among the conserved residues of all teleostean FXYD proteins studied, identical amino acids including phenylalanine (F29) of the FXYD motif, one tyrosine (Y33), and one glycine (G40) of the transmembrane domain were found (Tipsmark, 2008; Wang et al., 2008; Saito et al., 2010; Tang et al., 2012; Yang et al., 2013; Hu et al., 2014). In addition, structure prediction revealed that TnFXYD8 contains one transmembrane domain and a signal peptide (the first 18 amino acids). The signal peptides of mammalian FXYD1 and FXYD4, elasmobranch FXYD10, and certain teleost FXYD members were found to determine their orientation in the membrane. These FXYD members would yield mature proteins following cleavage of the approximately 20-amino acid N-terminal signal peptide (Mahmmoud et al., 2003; Geering, 2006; Tipsmark, 2008; Wang et al., 2008; Yang et al., 2013). Structural similarity indicated that TnFXYD8 might have similar functions to other known FXYD proteins in the modulation of NKA expression/activity.

Based on the results of the phylogenetic analysis, TnFXYD8 among these teleostean FXYD proteins was most similar to FXYD6, implying that they might have similar functions. Similar results were found in the other studies on teleosts (Tipsmark, 2008; Yang et al., 2013). In the Atlantic salmon, FXYD8 was considered to be a muscle-specific member because of its highest homology with the mammalian FXYD6 and tissue distribution (Tipsmark, 2008). However, unlike most FXYD proteins, *Tnfxyd8* was expressed ubiquitously in all organs studied in the pufferfish acclimated to either FW or SW. Similar results were found for the other FXYD proteins (Geering, 2006; Tipsmark, 2008; Saito et al., 2010; Yang et al., 2013). In mammals, FXYD5 was detected in a series of organs and tissues (Garty and Karlish, 2006; Geering, 2006). In Atlantic salmon, *fxyd*8 mRNA expression was also detected in several tissues including muscle (predominantly), gill, brain, and kidney (Tipsmark, 2008). Like the tissue distribution of *Tnfxyd8*, universal expression of *fxyd8* mRNA was found in the zebrafish (*Danio rerio*) (Saito et al., 2010) and medakas (Yang et al., 2013). Other FXYD proteins, such as *fxyd5* and/or *fxyd9*, were also expressed in various teleost tissues (Tipsmark, 2008; Saito et al., 2010; Yang et al., 2013). These results revealed that the roles of FXYD8 between the pufferfish and Atlantic salmon might be different because of their distinct tissue distribution. The ubiquitous expression of *Tnfxyd8* mRNA implies that it might be involved in various physiological reactions in different tissues. Among these tissues, the kidney is an important organ for osmotic homeostasis, especially in FW fish.

Previous studies have reported that an interaction exists between NKA α-subunit and FXYD proteins, and that NKA activity is modulated by this interaction (Béguin et al., 2001; Li et al., 2004; Geering, 2005). In the present study, the results of the immunoprecipitation assay indicated that an interaction exists between TnFXYD8 and the NKA α-subunit in the kidneys of pufferfish. Moreover, TnFXYD8 was co-localized with the NKA α-subunit in the renal tubules of the pufferfish, appearing in the basolateral membrane of epithelial cells in proximal, distal, and collecting tubules, but not in glomeruli (Lin et al., 2004). Similar results were found for branchial FXYD11 and renal FXYD12 in other teleosts. In gills, FXYD11 interacted with the NKA α-subunit in NKA-IR cells of the Atlantic salmon (Tipsmark et al., 2011), zebrafish (Saito et al., 2010), and brackish medaka (*Oryzias dancena*) (Yang et al., 2013). On the other hand, FXYD12 was also found to be expressed with the NKA α-subunit in the renal tubules of two medaka species (Yang et al., 2016b). Furthermore, salinity-dependent interactions were found for branchial FXYD11 and renal FXYD12 with the NKA α-subunit in the brackish medaka (Chang et al., 2016b; Yang et al., 2016b). These results suggested that teleostean FXYD proteins might be able to modulate NKA expression/activity via their interaction, as observed in mammals (Garty and Karlish, 2006; Geering, 2008).

There have been several investigations into the significance of renal NKA in the osmoregulation of euryhaline teleosts upon an osmotic challenge (Marshall and Grosell, 2006; Whittamore, 2012). In the pufferfish, significantly higher renal NKA activity, as well as α-subunit protein abundance, was found in FW- compared with SW-acclimated groups (Lin et al., 2004; Lin and Lee, 2016). When the pufferfish was transferred from FW to SW, renal NKA activity reduced within 3-h post-transfer (Lin and Lee, 2016). Those results indicate that the pufferfish is a euryhaline teleost with efficient osmoregulation, including NKA expression and activity for rapid ionic regulation and acclimation (Lin et al., 2004; Lin and Lee, 2016).

In contrast to NKA expression, the present study revealed that the protein expression of TnFXYD8 was significantly elevated in SW fish. Similar results were found in the pufferfish gill where SW or FW treatment also conversely regulated NKA and FXYD9 expression (Lin et al., 2004; Wang et al., 2008). Therefore, the different expression patterns of TnFXFYD8 and NKA may be related to the converse renal NKA activity between FW- and SW-acclimated pufferfish. In other teleostean kidneys, different patterns of FXYD expression and NKA activity were reported upon salinity challenge. A parallel pattern of FXYD12 protein and NKA activity was found in kidneys of the Japanese medaka (*O. latipes*) (Yang et al., 2016b). However, in the spotted scat (*Scatophagus argus*), there was no correlation between the expression patterns of renal *fxyd12* mRNA and NKA activity (Hu et al., 2014). In fish renal FXYD8, only *fxyd8* mRNA of the Atlantic salmon was investigated and showed no difference between FW and SW groups (Tipsmark, 2008).

To further elucidate the physiological functions of FXYD8 in fish, a functional assay of TnFXYD8 protein was conducted in *Xenopus* oocytes. Many studies have used the *Xenopus* oocytes system to explore the properties of FXYD proteins (Béguin et al., 2001, 2002; Crambert et al., 2005; Delprat et al., 2007). Herein, we found that overexpression of FXYD8 significantly decreased NKA activity in *Xenopus* oocytes. The finding may be resulted from the interaction between TnFXYD8 and endogenous NKA (i.e., direct effect) and/or from changes in endogenous NKA protein (i.e., indirect effect). Expression of endogenous NKA α- and β-proteins in *Xenopus* oocytes were demonstrated in a previous study (Béguin et al., 1997) and this study (Figure S1). Thus, the TnFXYD8 might interact the endogenous NKA α-subunits resulting in a low level NKA activity. On the other hand, previous studies indicated that presence or absence of FXYD protein might affect the NKA expression (isoform composition, protein abundance, and/or activity) (Garty and Karlish, 2006; Bibert et al., 2009; Saito et al., 2010). Therefore, the decrease of NKA activity might also result from the affected NKA protein. Regardless of the direct and/or indirect effects, a reduced NKA activity was found in the *Tnfxyd8*-cRNA injected oocytes. Hence, the results demonstrated that FXYD8 is able to negatively regulate NKA activity.

In fish, FXYD10 was found in shark only and its role in inhibition of NKA activity has been demonstrated (Mahmmoud et al., 2000, 2003). Positive correlations (mRNA and/or protein levels) between FXYD proteins and NKA have been commonly found, thus inferring that these FXYD proteins may induce NKA activity (Tipsmark, 2008; Saito et al., 2010; Tipsmark et al., 2010, 2011; Tang et al., 2012; Yang et al., 2013, 2016b; Chang et al., 2016b). For the functional assay, Saito et al. (2010) first analyzed FXYD11-knockdown in zebrafish and found an increased number of NKA-IR cells to compensate for the functional impairment of NKA. Similarly, on the basis of a FXYD12 knockdown experiment, FXYD12 was suggested to enhance NKA activity in kidneys of the brackish medaka (Yang et al., 2016b). Therefore, the potential roles of FXYD11 and FXYD12 were considered as positively modulating NKA activity (Saito et al., 2010; Yang et al., 2016b). In addition to NKA activity, in mammalian studies, FXYD proteins can modulate NKA by changing the apparent affinity of NKA for the sodium, potassium, and ATP (Geering, 2005, 2008). The phosphorylation states of FXYD proteins also have crucial impact in this process (Crambert and Geering, 2003; Garty and Karlish, 2006). In teleosts, these details of the modulatory mechanisms are still unknown and will be investigated in the future.

In summary, this study is the first to explore the physiological functions of FXYD8. Renal TnFXYD8 protein expression was significantly upregulated in SW-acclimated pufferfish and a protein-protein interaction was found to exist between FXYD8 and NKA in pufferfish kidneys. Furthermore, TnFXYD8 was found to exhibit an inhibitory effect on NKA expression/activity. The findings of the present study illustrate the potential functions of a novel NKA regulator in the kidneys of teleosts and further extend our understanding on the modulation of NKA activity by FXYD proteins in vertebrates.

## Author contributions

PW, CL, HH, and TL designed the experiments. PW and WY performed the experiments and analyzed the data. PW, WY, CL, and TL wrote the paper. All authors have read and approved the final manuscript.

### Conflict of interest statement

The authors declare that the research was conducted in the absence of any commercial or financial relationships that could be construed as a potential conflict of interest.
