# Supplementary material for: FXYD8, a Novel Regulator of Renal Na+/K+-ATPase in the Euryhaline Teleost, Tetraodon nigroviridis
Source: Front Physiol. 2017 Aug 8;8:576. doi: 10.3389/fphys.2017.00576 (PMC5550679; doi:10.3389/fphys.2017.00576)
Supplement: Supplementary file 1 [file DataSheet1.pdf]

<u>***Supplementary Information for***</u>

# FXYD8, a novel regulator of renal $Na^+/K^+$-ATPase in the euryhaline teleost, *Tetraodon nigroviridis*

Pei-Jen Wang[1,2†], Wen-Kai Yang[1,3†], Chia-Hao Lin[4], Hau-Hsuan Hwang[1], Tsung-Han Lee[1,5]*

*Corresponding author: T.H.L. thlee@email.nchu.edu.tw

## 1. Supplementary Methods

### S1. Immunolocalization

The kidney was excised and fixed immediately in methanol and dimethyl sulfoxide (4:1 v/v) at -20°C for 3 h. After washing with phosphate-buffered saline (PBS), the samples were mounted in optimal cutting temperature (OCT) compound (Tissue-Tek; Sakura, Torrance, CA, USA) at 4°C overnight and then mounted for cryosectioning. Sections were cut 5–7 µm thick using a Cryostat Microtome (Microm HM 505E; Walldorf, Germany), placed on 0.01% poly-L-lysine (Sigma, St. Louis, MO, USA) coated slides, and kept at -20°C before staining.

For double immunofluorescence staining, cryosections were rinsed with PBS and sequentially incubated in blocking buffer (5% BSA and 2% Tween 20 in PBS), anti-TnFXYD8 (5,000× dilution), secondary antibody (Alexa-Fluor-546-conjugated goat anti-rabbit antibody; Molecular Probes, Eugene, OR, USA), anti-NKA (100× dilution), and secondary antibody (Alexa-Fluor-488-conjugated goat anti-mouse antibody; Molecular Probes). The sections were mounted with coverslips using ClearMount Mounting Solution (Zymed, South San Francisco, CA, USA), and observed using a confocal laser scanning microscope (LSM510; Zeiss, Hamburg, Germany) with Zeiss LSM image software (**Figure S2**).

### S2. Overexpression of TnFXYD8 protein by bacterial strains

pET29a (Novagen, Madison, WI, USA) was used for *Tnfxyd8* overexpression in *Escherichia coli* (*E. coli*). The bacterial strains used in this work were *E. coli* DH5α and BL21 (DE3).

To generate the pET29a+*Tnfxyd8* construct, the corresponding TnFXYD8 coding region was PCR-amplified using plasmid pOSI-*Tnfxyd8* (pOSI-T vector carrying the *Tnfxyd8* gene) as the template with the following pairs of oligonucleotide primers: for TnFXYD8, 5'-CGGAATTCATGGACCTCGTGGTGTTTGT-3' (forward) and 5'-ATGCGGCCGCCTATTCTGCTTTGACAGAATAAG- 3' (reverse), and cloned into a pET29a vector at the EcoRI and NotI sites. The corresponding EcoRI, NotI and XbaI restriction sites are underlined at the primer described above. The sequences of all constructs were confirmed by DNA sequencing.

An overnight culture of BL21 (DE3) cells harboring pET29a+*Tnfxyd8* were diluted 20-fold in 10 mL Luria-Bertani medium containing 100 mg/L ampicillin and incubated at 37°C with 225 rpm shaking. When $OD_{600}$ of the culture reached 0.6, IPTG was added to a final concentration of 0.5 mM. After 2 h, the induced cells were harvested by centrifugation and used for immunoblotting detection of the TnFXYD8 protein (**Figures S3** and **S4**). According to the start codon of pET29a vector, the over-expressed protein revealed a higher molecular weight about 3.8 kDa than the predicted molecular weights of TnFXYD8.

## 2. Supplementary Tables

**Table S1. Primer sequences used for all PCR of pufferfish genes.**

| Gene | Primer sequence (5' to 3') | | Accession numbers | Amplicon Size (bp) |
|---|---|---|---|---|
| *Tnfxyd8* | Forward: | GTTCCAGGCGTAAAGAGTCG | HM585097, CR666825 | 146 |
| | Reverse: | GTCCAACAACTGCCTCCTTC | | |
| *β-actin* | Forward: | GTTGGAATGGGACAGAAGGA | CR733615 | 71 |
| | Reverse: | TTCAGCGTAAGGATGCCTCT | | |

**Table S2. Accession numbers of teleostean FXYD proteins.**

| Proteins | Accession numbers | Proteins | Accession numbers |
|---|---|---|---|
| Pufferfish (*Tetraodon nigroviridis*, Tn) | | Atlantic salmon (*Salmo salar*, Ss) | |
| TnFXYD8 | HM585097 | SsFXYD2 | DAA06140 |
| TnFXYD9 | EF028083 | SsFXYD5a | DAA06141 |
| | | SsFXYD6 | DAA06129 |
| Brackish medaka (*Oryzias dancena*, Od) | | SsFXYD7a | DAA06133 |
| OdFXYD5 | JX569227 | SsFXYD8 | DAA06130 |
| OdFXYD6 | JX624723 | SsFXYD9a | DAA06131 |
| OdFXYD7 | JX624724 | SsFXYD11a | DAA06135 |
| OdFXYD8 | JX569228 | SsFXYD12a | DAA06137 |
| OdFXYD9 | JX569229 | | |
| OdFXYD11 | JX624725 | Zebrafish (*Danio rerio*, Dr) | |
| OdFXYD12 | JX569230 | DrFXYD11 | NM_001282018 |
| | | | |
| Japanese medaka (*Oryzias latipes*, Ol) | | Japanese eel (*Anguilla japonica*, Aj) | |
| OlFXYD5 | ENSORLT00000008380 | AjFXYD11 | AFK24487 |
| OlFXYD6 | ENSORLG00000007198 | | |
| OlFXYD7 | JX565424 | Spotted scat (*Scatophagus argus*, Sa) | |
| OlFXYD8 | JX565423 | SaFXYD11 | KF649216 |
| OlFXYD9 | JX565422 | SaFXYD12 | KF649215 |
| OlFXYD11 | JX624726 | | |
| OlFXYD12 | JX643983 | | |

The "ENSORL" prefix indicates accession numbers from the Ensembl (http://asia.ensembl.org/); other letters indicate accession numbers from the National Center for Biotechnology Information (http://www.ncbi.nlm.nih.gov/).

### 3. Supplementary Figures

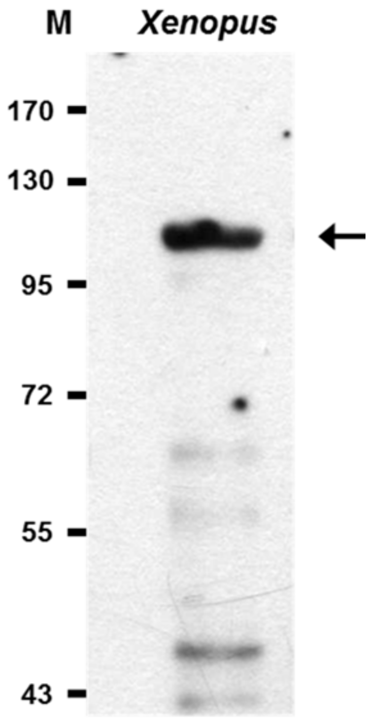


**Figure S1. NKA detection of *Xenopus* oocyte protein lysate revealed immunoreactive bands at 105 kDa (arrow).** M, marker (kDa).

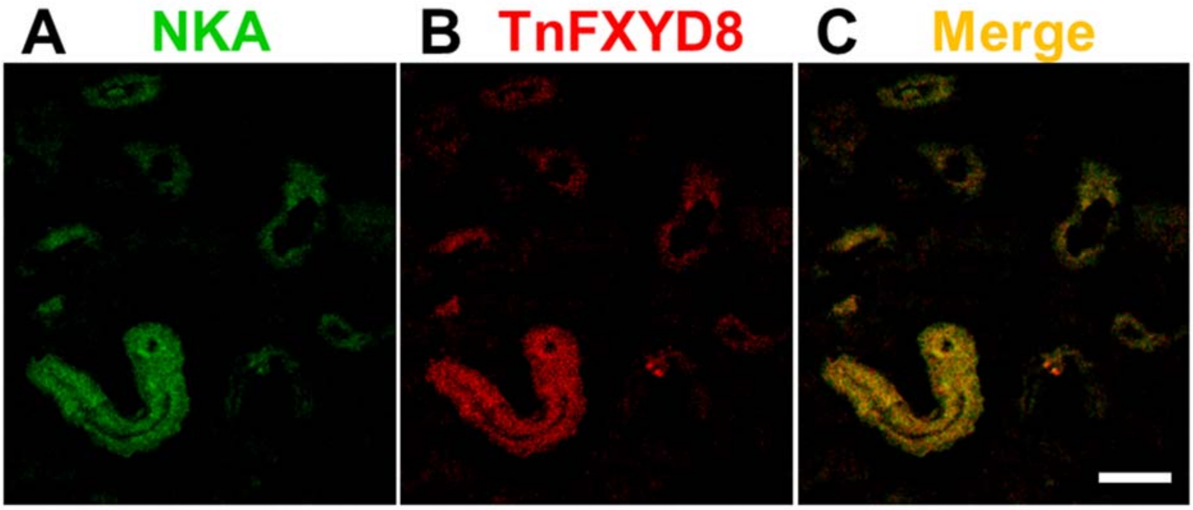


**Figure S2. Immunolocalization of $Na^+/K^+$-ATPase (NKA; green; A) and TnFXYD8 protein (red; B) in frozen sections of the kidneys of seawater-acclimated pufferfish.** (c) Merged image of the confocal microscope revealed that NKA and TnFXYD8 were co-localized in the basolateral membrane of epithelial cells in renal tubules. Scale bar, 50 μm.

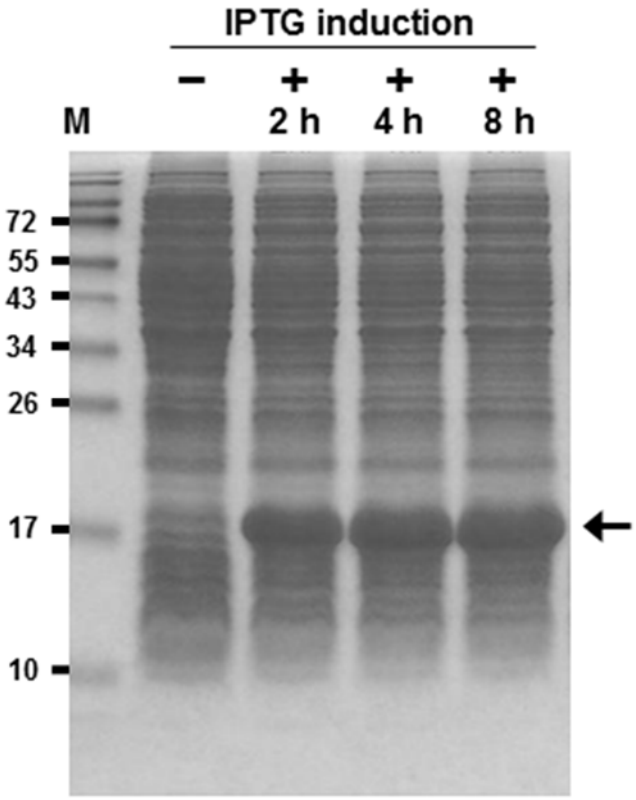


**Figure S3. Coomassie blue staining of TnFXYD8 overexpression in bacteria lysate.** Bacteria lysates were separated by sodium dodecyl sulfate-polyacrylamide gel. TnFXYD8 overexpression protein were detected at 17 kDa after IPTG 2 h induced (arrow). M, marker (kDa); −, non-induction; +, over expression.

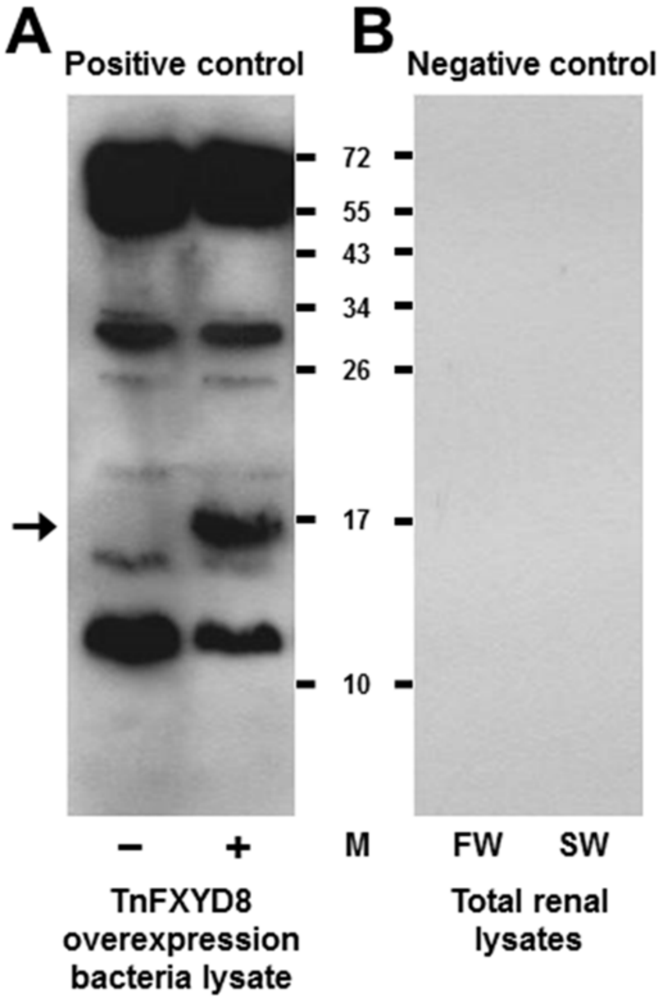


**Figure S4. Specificity analysis of the TnFXYD8 antiserum.** TnFXYD8 overexpressed bacteria lysate (**A**; positive control) and total renal lysates (**B**; negative control) were separated by SDS-polyacrylamide gel electrophoresis and transferred to PVDF membrane. The additional immunoreactive band at 17 kDa was detected by TnFXYD8 antiserum in TnFXYD8 overexpression bacteria lysate (arrow). The pre-immune serum was used as the negative control which revealed no immunoreactive band. +, over expression; −, non-induction; M, marker (kDa); FW, fresh water; SW, seawater.
